# Macrophage Migration Inhibitory Factor Gene Polymorphism in Acute Coronary Syndrome

**DOI:** 10.7759/cureus.69914

**Published:** 2024-09-22

**Authors:** Likitha M J, Sharan Badiger, Gurushantappa S Kadakol

**Affiliations:** 1 Medicine, Shri B. M. Patil Medical College Hospital and Research Centre, BLDE (Deemed to Be University), Vijayapura, IND; 2 Genetics, Shri B. M. Patil Medical College Hospital and Research Centre, BLDE (Deemed to Be University), Vijayapura, IND

**Keywords:** acute coronary syndrome, genetic polymorphism, genetic study, macrophage migration inhibitory factor, major adverse cardiac event

## Abstract

Background

Acute coronary syndrome (ACS) is a result of the interplay between genetic and environmental risk factors. A unique cytokine macrophage migration inhibitory factor (MIF), expressed by inflammatory cells, acts via a cluster of differentiation 74 (CD74) and a cluster of differentiation 44 (CD44) receptors, leading to the recruitment of mononuclear neutrophils and lymphocytes. This cascade results in exaggerated inflammation and atherosclerosis. MIF's distinctive characteristics and functions make it an essential target for achieving therapeutic atherosclerosis regression, setting it apart from other cytokines. Hence, this study aims to detect the MIF gene polymorphism in ACS patients and to assess the incidence of in-hospital major adverse cardiac events (MACE).

Methodology

This study was conducted in a tertiary care hospital. A total of 90 patients who had ACS were enrolled, of which 83 were included, and seven patients were excluded based on exclusion criteria (four cases of old myocardial infarction, two cases of valvular heart disease, and one case of dilated cardiomyopathy). After detailed clinical examination, laboratory evaluation, and genetic test, patients were divided into two groups based on MIF gene mutation. Five patients who had positive MIF gene mutation were termed as group A (n=5), and 78 patients with negative MIF gene mutation were termed as group B (n=78). The statistical analysis for the collected data was done using IBM SPSS Statistics for Windows, Version 20 (Released 2011; IBM Corp., Armonk, New York, USA).

Results

Out of the 83 patients in this study, the male gender was predominant; there were three male patients in group A (n=3; 60%) and 48 male patients in group B (n=48; 61.5%). The most common age group was between 60 and 69 years; two of five patients (n=2; 40%) in group A and 30 out of 78 patients (n=30; 38.4%) in group B belonged to this age group. The common symptom was chest pain present in five patients in group A (n=5; 100%) and 76 patients (n=76; 97.5%) in group B. A common risk factor in group A patients was tobacco chewing, seen in three patients (n=3; 60%), and group B smoking was the most common risk factor seen in 30 patients (n=30; 38.5%). The most common ECG finding in group A was ST-elevation myocardial infarction (STEMI), seen in three patients (n=3; 60%), and in group B, the commonest ECG finding was non-ST-elevation myocardial infarction (NSTEMI) seen in 23 patients (n=23; 29.5%). The most common MACE was heart failure seen in two patients in group A (n=2; 40%) and in 50 patients in group B (n=50; 64.1%), followed by arrhythmias seen in one patient in group A (n=1; 20%), and eight patients in group B (n=8; 10.3%).

Conclusion

This study demonstrated a significant positive association between MIF gene polymorphism and the occurrence of cardiovascular events like myocardial infarction, with a statistically significant p-value (p=0.001). This study showed the presence of the disease in young age groups and individuals without conventional risk factors, underscoring the importance of genetic studies. Genetic risk factors independently contribute to the pathophysiology of coronary artery disease; hence, understanding the mechanisms of these genes and incorporating genetic testing into standard clinical practice can help future research to develop therapeutic agents that can specifically target these genes.

## Introduction

According to a survey conducted by the Global Burden of Disease (GBD), it was found that in India, more than 272 per one lakh deaths are attributed to cardiovascular diseases, which is higher than the death rate reported in the entire world [1]. One of the common reasons for the occurrence of acute coronary syndrome (ACS) is the occlusion of coronary blood flow to the myocardium due to the presence of atheromatous plaque within these blood vessels. It is primarily the result of the interaction between hereditary and environmental risk factors, including modifiable risk factors like sedentary lifestyle, habits, and metabolic abnormalities, and non-modifiable risk factors like age, sex, and genetic mutations [2]. An acute myocardial infarction is a general phrase that refers to the death of cardiomyocytes that occurs due to protracted ischemia brought on by an abrupt imbalance between the supply and demand of oxygen [3]. Even though several risk factors play a significant part in the disease's evolution, atherosclerosis ultimately results in the creation of atheroma and the obstruction of blood vessels [4]. Hypercholesterolemia, the best-known risk factor for atherogenesis, leads to early endothelial dysfunction by activating adhesion molecules and subintimal mononuclear cell infiltration, the first morphological sign of arterial inflammation [5]. Variants of this include myocardial infarction with non-obstructive coronary arteries (MINOCA), where plaque disruption occurs in the absence of significant stenosis, and it carries a better prognosis compared to conventional ACS [6].

A cytokine known as macrophage migration inhibitory factor (MIF) plays a role in various pathological processes of cardiovascular illness; its expression is linked to increased thickness of vessel walls, excessive deposition of lipids within the vessels, and severe forms of atherosclerotic disease [7]. More knowledge of the molecular machinery behind these effects has only recently been brought to light after finding that the chemokine receptors CXCR2 and CXCR4 are functional receptors for MIF [8]. MIF can initiate a calcium influx that quickly activates integrins, mediating the G-α receptors and integrin-dependent arrest as well as monocyte and T-cell chemotaxis [9]. Reactive oxygen species (ROS) produced within the cardiomyocytes lead to the activation of multiple genes, including MIF [10]. The ROS results in extracellular signal-regulated kinase activation, causing cardiomyocytes to secrete MIF via the protein kinase C-dependent export mechanism [11].

During the course of atherosclerosis, oxidized low-density lipoprotein and angiotensin II induce the production of MIF, which is expressed in both the coronary artery walls and damaged myocardial cells. Following its release, MIF upregulates a number of inflammatory mediators, including T lymphocytes, monocytes, vascular cell adhesion molecules (VCAM), endothelial cell adhesion molecules (ECAM), and chemokines. This increased inflammation within the arterial wall causes the development of atherosclerotic plaque. MIF also causes degradation of elastin and collagen within the plaque, which is responsible for unstable plaque formation and plaque rupture; this goes on to block the coronary vessels, resulting in acute myocardial infarction [12].

## Materials and methods

This cross-sectional study was conducted on patients with ACS from December 2022 to February 2024 in Shri B. M. Patil Medical College Hospital and Research Centre, BLDE (Deemed to Be University), Vijayapura, India. An institutional ethical clearance certificate with reference number (IEC/748/2022-23) was obtained. Additionally, the study was registered with the Clinical Trial Registry India (CTRI/2022/11/047723). The sample size was calculated to predict a mean with a 95% confidence interval and precision of 0.13 using the t-distribution. The inclusion criteria were all patients presenting with ACS (ST-segment elevation MI, non-ST-segment elevation MI, unstable angina), and the exclusion criteria were patients with a history of previous myocardial infarction, which was confirmed by old medical records, valvular heart disease, and cardiomyopathy. Since patients with these conditions might have already developed complications due to the primary disease and the occurrence of in-hospital major adverse cardiac events (MACE) cannot be attributed purely to ACS, this would affect the data interpretation; hence, these conditions have been excluded from the study. All patients underwent standardized assessment with detailed history, clinical examination, and investigations like cardiac enzymes, including creatine phosphokinase-MB (CPK-MB), Troponin I by chemiluminescence immunoassay method, and electrocardiogram on admission. Other relevant investigations, such as lipid profile, renal profile, and echocardiography, were also performed. Overall, 90 patients were enrolled in the study, of which 83 patients with ACS were included after taking prior informed and written consent. Seven out of 90 patients were excluded who fit the exclusion criteria (four of them had a history of old myocardial infarction, two had valvular heart disease, and one had cardiomyopathy). One ml of peripheral blood sample from 83 included patients was taken for analysis of MIF gene polymorphism, and further coronary angiography (if required) was done.

Detection of MIF polymorphism

After blood collection and storage, primers were designed for the target region of the MIF gene by using the bioinformatics tool NCBI with reference sequence NG_012099.1 as shown in Table 1.

**Table 1:** Primer details for MIF gene MIF: migration inhibitory factor

Forward primer	Reverse primer	Product size	Tm
TTGCACCTATCAGAGACC	TCCACTAATGGTAAACTCG	211	56^0^

Genomic DNA samples were subjected to a conventional polymerase chain reaction (PCR). A total of 20 μl final volume containing 1 μl of DNA (20 ng/μl to 100 ng/μl), 10 μl of readymade master mix containing 0.4 μl of deoxynucleotide triphosphate (dNTP) (10 pmol), 0.2 μl of Taq DNA polymerases (3 U/μl), 4 μl of Taq buffer (5×) (Takara, Japan), 0.4 μl of each primer (5 pmol), and the final volume was adjusted up to 20 μl using nuclease-free water. These prepared PCR reactions have been carried out in a thermocycler (Virit, Thermo Scientific, USA) under the following PCR cycle conditions: 95°C for five minutes for initial denaturation, followed by 35 cycles at 95°C for 30 seconds (cycle denaturation), 56°C for 30 seconds for primer annealing, 72°C for one minute for elongation, and 72°C for five minutes for final extension. These products were subjected to 2% agarose gel electrophoresis with a 100-bp DNA ladder to confirm the end products of PCR. Using the variant reporter software (ABI v1.1), the produced sequences were aligned to the corresponding reference sequences, and sequences were screened to look for familiar variants like deletions, insertions, and new mutations.

Statistical analysis

We analyzed all the data using the IBM SPSS Statistics for Windows, Version 20 (Released 2017; IBM Corp., Armonk, New York, USA). We used the chi-square test for qualitative variables and the Mann-Whitney U test to compare the continuously distributed quantitative variables that were normally distributed between the two groups. We presented the results using percentages, mean ± SD, and tables. A p-value of less than 0.05 was considered statistically significant, and all statistical tests were performed two-tailed.

## Results

A total of 83 ACS patients were included in this study. Based on the presence or absence of MIF gene mutation, these patients were divided into group A (n=5) and group B (n=78), respectively. In group A there were three male (n=3; 100%) and two female patients (n=2; 100%), and in group B there were 48 male patients (n=48; 100%) and 30 female patients (n=30; 100%). The common age group in group A was 31 to 40 years; one male patient (n=1; 33.3%); and one female patient (n=1; 50%) belonged to this age group, followed by the age group of 51 to 60 years; one male patient (n=1; 33.3%) and one female patient (n=1; 50%) in group A belonged to this age group. In group B, the most common age group was 61 to 70 years; 21 male patients (n=21; 43.7%) and nine female patients (n=9; 30.3%) belonged to this age group. The demographic and clinical data of all 83 patients is summarized in Table 2.

**Table 2:** Demographic and clinical data ^*^p<0.05, statistically significant

Demographic data	Group A (n=5)		Group B (n=78)		Chi-square value	p-value
Age group	Male (n=3) (%)	Female (n=2) (%)	Male (n=48) (%)	Female (n=30) (%)	11.86	^*^0.018
21-30 years	0 (00.0%)	0 (00.0%)	0 (00.0%)	0 (00.0%)		
31-40 years	1 (33.3%)	1 (50.0%)	2 (4.17%)	1 (3.33%)		
41-50 years	0 (00.0%)	0 (00.0%)	5 (10.4%)	2 (6.67%)		
51-60 years	1 (33.3%)	1 (50.0%)	8 (16.6%)	0 (0.00%)		
61-70 years	1 (33.3%)	0 (00.0%)	21 (43.7%)	9 (30.3%)		
>70 years	0 (00.0%)	0 (00.0%)	12 (25.0%)	6 (20.0%)		
Clinical data						
Chest pain	3 (100%)	2 (100%)	46 (95.8%)	30 (100%)	1.080	0.782
Dyspnea	0 (00.0%)	1 (50.0%)	18 (37.5%)	20 (66.6%)	1.568	0.457
Abdominal pain	0 (00.0%)	0 (00.0%)	2 (4.17%)	3 (10.0%)	0.341	1.000
Palpitation	0 (00.0%)	0 (00.0%)	4 (8.3%)	2 (6.6%)	0.415	0.680
Diabetes	2 (66.6%)	1 (50.0%)	12 (25.0%)	8 (26.6%)	1.852	0.396
Hypertension	1 (33.3%)	2 (100%)	10 (20.0%)	10 (33.3%)	3.124	0.373
Smoking	2 (66.6%)	0 (00.0%)	30 (62.5%)	0 (00.0%)	0.076	0.963
Alcohol	1 (33.3%)	0 (00.0%)	15 (31.2%)	0 (00.0%)	0.075	0.963
Tobacco chewing	1 (33.3%)	2 (100%)	8 (16.0%)	4 (13.3%)	6.317	0.039

Chest pain was present in all five patients of group A: three male patients (n=3; 100%) and two female patients (n=2; 100%), and in group B, 46 of 48 male patients (n=46; 95.6%) and 30 of 30 female patients (n=30; 100%) had chest pain, making it the most common presenting complaint in both groups. In group A, tobacco chewing was the most common risk factor; it was seen in one of three male patients (n=1; 33.3%) and two of two female patients (n=2; 100). Smoking was the most common risk factor in group B, seen in 30 of 48 male patients (n=30; 62.5%) and in zero female patients. The laboratory data of patients from both groups has been depicted in Table 3 along with their p-values.

**Table 3:** Hemodynamic and laboratory data RBS: random blood sugar

Hemodynamic and laboratory data	Reference range	Group A (n=5)		Group B (n=78)		Mann Whitney-U values	p-value
		Mean	SD	Mean	SD		
Pulse rate (beats per minute)	80-100	85.20	12.69	60.46	9.763	152.0	0.115
Temperature (degree Celsius)	36.5-37.5	37.8	1.234	39.0	1.445	163.0	0.348
Hemoglobin (g/dl)	13-17	12.44	1.073	12.37	2.555	174.0	0.688
Total count (10^^^3/ml)	4-10	37.8	1.234	39.0	1.445	146.0	0.348
RBS (mg/dl)	70-140	129.6	35.816	35.816	46.670	186.0	0.863
Blood urea (mg/dl)	19-43	26.80	5.167	36.473	20.906	148.50	0.373
Serum creatinine (mg/dl)	0.4-1.1	0.820	0.2280	1.119	0.6643	131.50	0.222
Serum sodium (mmol/l)	135-145	139.6	4.775	137.9	5.534	167.0	0.591
Sr. potassium (mmol/l)	3.5-5.1	3.980	0.5762	4.073	0.719	180.0	0.774
Total cholesterol (mg/dl)	<200	173.6	20.659	174.9	32.92	194.0	0.985
Triglycerides (mg/dl)	<150	140.8	83.497	113.7	27.69	188.0	0.893
Troponin I (pg/ml)	<11.6	3692.5	4970	4768	7671	177.50	0.771

**Figure 1:**
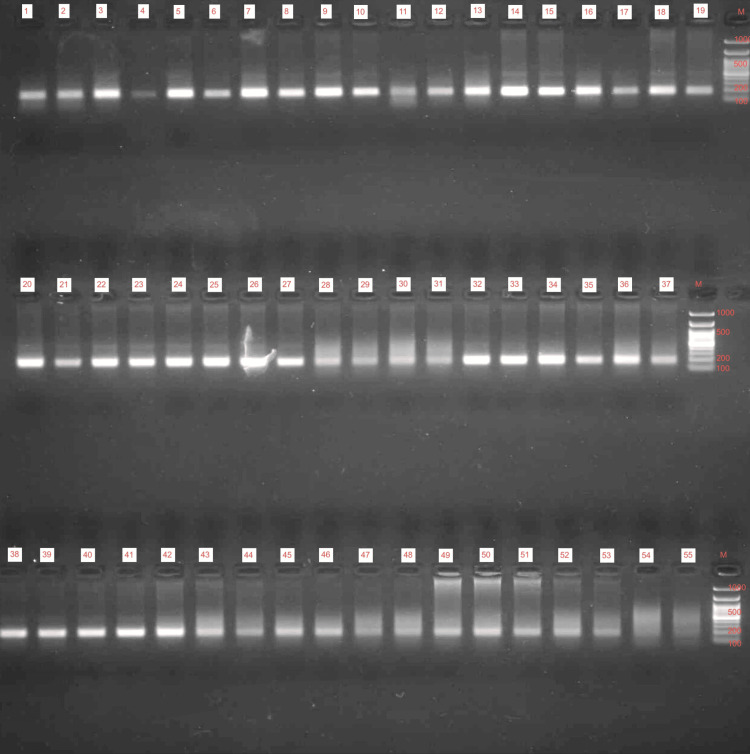
Polymerase chain reaction products Amplification of DNA products using polymerase chain reaction technique DNA: deoxyribonucleic acid

Out of the 83 patients analyzed for MIF gene mutation, only five patients showed a positive association for CAAT 6; this variant refers to a specific polymorphism in the promoter region of MIF where the tetra nucleotide sequence CATT is repeated six times. The PCR products are shown below in Figure 1, and the distribution of patients with MIF-positive mutations is shown in Table 4.

**Table 4:** Distribution of macrophage migration Inhibitor factor gene (MIF) polymorphism ^*^p<0.05, statistically significant

MIF gene mutation	Group A (n=5)		Group B (n=78)		Chi-square value	p-value
	No. of patients	Percentage	No. of patients	Percentage		
Present	5	100.0%	0	00.0%	83.000	^*^0.001
Absent	0	00.0%	78	100.0%		
Total	5	100.0%	78	100.0%		

Further, all 83 patients were assessed for MACE; heart failure was the most common MACE in both groups, seen in two patients in group A (n=2; 40%) and 50 patients in group B (n=50, 64.1%). Further in group A, one patient had arrhythmia (n=1; 20%), and one patient had in-hospital death (n=1; 20%). In group B, eight patients had arrhythmia (n=8; 10.2%), 10 patients had pulmonary edema (n=10; 12.8%), one patient had cardiogenic shock (n=1; 1.3%), and one patient had in-hospital death (n=1; 1.3%).

## Discussion

Various studies from all over the world have been conducted to know the genetic polymorphism in coronary artery disease; this study, however, mainly includes the Vijayapura Karnataka population and has attempted to screen the presence of MIF polymorphism in these patients. In this study, most patients were aged above 60 ± 10 years (p=0.018). A similar study done by Rosengren et al. in the year 2006 on 10253 ACS patients across 103 hospitals in Europe showed that most patients were aged between 55 and 75 years [13], which implies that increasing age poses a significant risk for developing MI. Old age is a risk factor that is associated with both ACS and MIF gene polymorphism because, as age increases, it accelerates the process of atherosclerosis and plaque formation, which in turn facilitates the release of MIF into the plasma and amplifies the events of plaque rupture and acute MI.

This study showed male predominance; three of five patients in group A (n=3; 60%) and 48 out of 78 patients in group B (n=48; 61.5%) were males, which resembles a study done by Sharma et al. in 2014 in Jayadeva Institute of Cardiology, Bangalore, where they studied 1562 patients with ACS in which the majority of them were male (1242 (79.5%)) and 320 (20.5%) were females [14]. In all 83 individuals, this study examined modifiable risk factors such as alcohol intake, smoking, tobacco chewing, hypertension, and diabetes, along with non-modifiable risk factors like age and gender. Tobacco chewing was the commonest risk factor in group A patients who had a positive MIF mutation, and it was statistically significant (p=0.039). Zhang et al. studied 963 ACS patients in China in the year 2021, in which they showed that risk factors like smoking (p=0.001), diabetes, and hypertension were commonly associated with ACS [15]. Tobacco chewing (smokeless tobacco) is a common and gender-neutral practice in this geographical region when compared to Western countries where smoking is more common. According to a review article by Gupta et al. on smokeless tobacco and cardiovascular risk, it was found that smokeless tobacco has adverse effects on lipid profiles; they have an increased risk of hypercholesterolemia. N-nitrosamines and other alkaloids found in tobacco produce extremely reactive electrophiles and raise oxidative stress. It has been demonstrated that in vitro, smokeless tobacco extract is more harmful than pure nicotine and increases oxidative stress due to reactive oxygen free radicals. Also, due to high fibrinogen levels, the risk of MI and stroke is higher in tobacco chewers [16]. These studies suggest that lifestyle modification and habit changes may be beneficial in the reduction of these risk factors. Out of 83 patients with ACS in this study, the most common symptom was chest pain, followed by dyspnea and palpitations. In a study done by O'Donnell et al. in 2021 in Ireland with 1947 ACS patients, they reported that chest pain was the most common symptom seen in 71% of patients [17]. In this study of 83 patients, group A had five patients, three males and two females, who showed positive mutation for the MIF gene; they showed specific polymorphism in the promoter region, where the tetra nucleotide sequence "CATT" is repeated six times (CATT 6); this was statistically significant (p=0.001). In a clinical trial conducted by Yüksel et al., between July 2012 to March 2013, a total of 87 patients presenting with chest pain were evaluated of which 65 had ACS and were taken as cases and the rest 22 had non-cardiac chest pain were taken as controls, the cases were further subdivided into STEMI (n=30) and NSTEMI (n=35) group, in their study they aimed to evaluate the plasma MIF and E-selectin levels in patients with ACS (cases) and controls, they concluded that there was a significant increase in plasma MIF levels in cases than in controls; however, the plasma E-selectin levels was similar in both cases and control group, their results suggest that MIF may play a role in the causation of atherosclerosis and MI [18]. In this study, a specific polymorphism of the MIF gene in the promoter region where the tetra nucleotide sequence “CATT” repeated six times (CATT 6) was identified and this is significant because the length of the repetitions and the expression of the genetic marker are correlated; higher alleles (CATT6, CATT7, and CATT8) exhibit higher gene expression as reported by Valdés-Alvarado et al. in the study conducted in the year 2014 [19]. Another study by Pereira et al. conducted a prospective study in the USA in the year 2005, where within 12 hours of admission, a peripheral blood sample was taken for serum CD40L, MIF, and IL-6 tests. About 35.4% (69 out of 195) and 64.6% (126 out of 195) of individuals were deemed to be at high risk for ACS and non-ACS groups, respectively. Serum CD40L and MIF showed a positive bidirectional association with the total number of patients, while each variable showed a negative correlation with IL-6 [20].

Further in this study, the occurrence of in-hospital MACE was evaluated, and heart failure was found to be the most common MACE, followed by arrhythmia, pulmonary edema, cardiogenic shock, and in-hospital deaths. In a study done by Liu et al. aimed to study the new genetic variants associated with MACE in ACS patients, conducted in China from 2009 to 2012 with a sample size of 1916 patients with ACS, they identified eight genes, e.g., MYOM2, WDR24, etc., in association with MACE [21]. A study by Borkowski et al. in 2024 reviewed the socioeconomic and racial determinants of cardiovascular diseases and came to the conclusion that low socioeconomic status, insufficient insurance coverage, and a lack of food and housing security all contribute to poor disease outcomes [22]. The occurrence of MACE not only depends on the severity of the disease but also on other factors like the availability of appropriate treatment on time and observation in the intensive care unit (ICU) to prevent any further events. These factors are influenced by the socioeconomic status of the patients. In this study, most patients belonged to low socioeconomic backgrounds, which might be a challenging aspect to overcome.

All the above-mentioned studies conducted in the Southeast Asian population and the USA have demonstrated the significant role of MIF gene polymorphism in atherosclerosis and the causation of ACS. However, no such study has been conducted in southern India, and this is one of the novel studies conducted in the Vijayapura population. Since genetic risk factors independently contribute to the pathophysiology of coronary artery disease, understanding the mechanisms of these genes and incorporating genetic testing into standard clinical practice can aid in the development of therapeutic agents that specifically target these genes. Through the identification of asymptomatic high-risk relatives of ACS patients, genetic advancements can aid in the achievement of primary prevention of the disease. One such attempt has been made in this study by demonstrating MIF gene polymorphism in ACS patients.

Limitation

This study has a small sample size and represents a population of a single area of Southern India; hence, the positive correlation of the MIF gene obtained in this study with ACS patients cannot be generalized. Given the small sample size, this might affect the statistical tests. Expanding the sample size in future studies by screening the family members at risk could offer deeper insights into the role of MIF polymorphisms in ACS research. The need for specialized laboratory equipment in genetic studies limits its accessibility, as it is only available in urban settings and to patients with a higher socioeconomic background.

## Conclusions

This study attempts to detect MIF gene polymorphism in ACS patients as MIF plays a pivotal role in atherosclerosis, which is the main culprit leading to coronary artery disease. The study showed a statistically significant positive correlation (p=0.001) between the MIF gene polymorphism and the incidence of cardiovascular events such as myocardial infarction. This study also noted the occurrence of in-hospital MACE and its correlation with positive MIF gene mutation. The relevance of genetic research is shown by this study, which revealed the disease's occurrence in people without traditional risk factors and in young age groups. The pathophysiology of coronary artery disease is partly influenced by genetic risk factors, and its diagnosis can be difficult at times due to the disease's complex mechanism and presentation. To overcome these challenges, gene analyses might be helpful. Hence, this study can add value to future research on the role of genetics in cardiovascular diseases.
